# The Efficacy of Dexmedetomidine Versus Ketamine for Sedation in Pediatric Dental Procedures: A Systematic Review and Meta-Analysis

**DOI:** 10.3390/children13040558

**Published:** 2026-04-17

**Authors:** AbdulRahman Alasmri, Ali Alhijab, Shahad N. Abudawood, Narmin Hilal, Heba Jafar Sabbagh

**Affiliations:** Pediatric Dentistry Department, Faculty of Dentistry, King Abdulaziz University, P.O. Box 80200, Jeddah 21589, Saudi Arabia

**Keywords:** pediatric dentistry, sedation, dexmedetomidine, ketamine, dental anxiety, systematic review, meta-analysis, behavior management

## Abstract

**Highlights:**

**What are the main findings?**

- Dexmedetomidine and ketamine provided similar analgesic effectiveness during and after dental procedures in children.
- Pooled analyses showed no statistically significant differences between dexmedetomidine and ketamine in heart rate, oxygen saturation, or recovery time, though evidence certainty was low.

**What are the implications of the main findings?**

- Both agents appear viable for pediatric dental sedation based on limited evidence; Dexmedetomidine showed significantly lower systolic blood pressure; larger trials are needed to confirm clinically meaningful differences.
- To improve clinical guidelines for sedation, there is a need for larger, standardized randomized trials.

**Abstract:**

**Background:** Effective and safe sedation is essential in pediatric dental practice to manage anxiety, pain, and cooperation during procedures. **Objective:** This exploratory systematic review and meta-analysis aimed to synthesize available RCT evidence comparing dexmedetomidine and ketamine across different sedation strategies (premedication and procedural sedation) in children undergoing dental procedures. **Methods:** This study was conducted and reported in accordance with the PRISMA 2020 statement. A comprehensive literature search was performed across PubMed, Web of Science, Scopus, and ScienceDirect for studies between 1990 and 2026. Randomized controlled trials (RCTs) were included. The primary outcomes assessed were intraoperative and postoperative analgesia, heart rate, systolic and diastolic blood pressure (SBP and DBP), oxygen saturation (SpO_2_), and recovery time. A meta-analysis of the extracted data was performed, and the risk of bias was assessed using the Cochrane risk of bias tool 2. **Results:** The review included four RCTs involving 178 children, with a mean age of 6.5–9.1 years. Intraoperative and postoperative analgesia did not differ significantly between groups (*p* = 0.09 and *p* = 0.08, respectively). Pooled analysis showed numerically lower heart rates with dexmedetomidine compared to ketamine, but the difference was not statistically significant (MD = −11.70; 95% CI: −29.27 to 5.86; *p* = 0.07). Systolic blood pressure was significantly lower with dexmedetomidine (MD = −6.69; 95% CI: −6.91 to −6.47; *p* = 0.002). Oxygen saturation did not differ significantly between groups (*p* = 0.35). Two studies were rated as having a low risk of bias. The remaining two had some concerns, related to unverified blinding and lack of outcome pre-specification. **Conclusions:** Based on limited and clinically heterogeneous evidence, both dexmedetomidine and ketamine appear to be viable options for sedation in pediatric dental settings, though no firm superiority conclusion can be drawn. Further randomized controlled trials with larger sample sizes and standardized sedation protocols are recommended to strengthen these findings and inform practice guidelines.

## 1. Introduction

Dental fear and anxiety (DFA) represent common challenges in pediatric dentistry and may significantly interfere with the successful delivery of dental care. Children experiencing high levels of anxiety may demonstrate behavioral resistance, distress, or refusal of treatment, which can compromise both treatment quality and patient safety. The prevalence of dental anxiety among children varies widely and has been estimated to affect approximately 30% of pediatric patients [1], with variations influenced by age, gender, previous dental experiences, and socio-cultural background [2,3]. Notably, the COVID-19 pandemic may have further influenced pediatric dental anxiety, altered treatment demand, and affected sedation practice patterns. The pandemic disrupted the daily practice of dental professionals, with significant economic, managerial, and psychological implications [4]. Furthermore, delays in routine dental care during the pandemic period may have increased the number of children presenting with advanced caries requiring sedation for treatment [2]. This evolving clinical landscape reinforces the need for evidence-based guidance on sedation agent selection.

Pain and anticipation of discomfort are among the primary contributors to dental anxiety and may negatively influence a child’s cooperation during dental procedures. This challenge is particularly evident in children with early childhood caries (ECC), who frequently require extensive dental treatment at a young age. Due to their limited cognitive and emotional development, these patients often exhibit uncooperative or distressed behaviors that complicate treatment delivery [5].

Behavior guidance is therefore a cornerstone of pediatric dental practice. According to the American Academy of Pediatric Dentistry (AAPD), behavior management strategies range from basic non-pharmacological approaches to advanced pharmacological techniques when necessary [6]. Techniques such as Tell-Show-Do and positive reinforcement are commonly used and effective in many cases; however, they may be insufficient for children with severe anxiety, extensive treatment needs, or limited coping ability [7]. In such situations, pharmacological sedation becomes an essential adjunct to facilitate safe and effective dental care.

Among the pharmacological agents used for pediatric procedural sedation, ketamine and dexmedetomidine have received increasing attention. Ketamine is a dissociative anesthetic that provides sedation, analgesia, and amnesia while maintaining airway reflexes and spontaneous respiration, making it widely used in pediatric procedural settings. Dexmedetomidine, a highly selective α2-adrenoceptor agonist, produces sedation and anxiolysis with minimal respiratory depression and has emerged as a promising alternative sedative agent in pediatric patients [8,9].

Despite their growing use in pediatric dentistry, the comparative effectiveness and safety of dexmedetomidine and ketamine remain unclear, particularly in dental procedural settings. Existing studies provide heterogeneous findings, and a comprehensive synthesis of the available evidence is lacking.

To our knowledge, existing meta-analytic evidence directly comparing dexmedetomidine and ketamine in pediatric dental settings remains limited. This gap in adequately synthesized data restricts evidence-informed comparative recommendations.

The purpose of this exploratory systematic review and meta-analysis was to consolidate existing randomized controlled trial (RCT) data evaluating dexmedetomidine versus ketamine in various pediatric dental sedation scenarios. The analysis emphasized surrogate measures of sedation quality, including analgesic effectiveness, hemodynamic stability, respiratory safety, and recovery profiles.

## 2. Materials and Methods

The Preferred Reporting Items for Systematic Reviews and Meta-Analyses (PRISMA) guidelines [10] were followed, and the findings were reported in accordance with the PRISMA statement [10].

### 2.1. Registration

We registered our study in the International Prospective Register of Systematic Reviews (PROSPERO) (https://www.crd.york.ac.uk/prospero/) (accessed on 15 January 2026), with registration number CRD42024531583.

### 2.2. Information Sources

All relevant studies published between 1990 and 2026 were identified. A comprehensive search of electronic databases (PubMed, Web of Science, Scopus, and ScienceDirect) was conducted for studies published between January 1990 and 15 January 2026. The search date range was chosen because dexmedetomidine was first introduced clinically in 1999, and the broad starting date ensured capture of any early comparative studies. A manual search of reference lists from identified published works and Google Scholar was also conducted to identify potentially eligible studies. A combination of Medical Subject Headings (MeSH) terms (for PubMed) and free-text keywords (for other databases) was used. Duplicate studies were identified and removed using the EndNote reference manager (version 21, Niles Software, Philadelphia, PA, USA).

### 2.3. Search Strategy

The search was updated on 15 January 2026. The full PubMed search strategy was: ((“Dexmedetomidine”[MeSH Terms] OR “dexmedetomidine”[Title/Abstract] OR “precedex”[Title/Abstract]) AND (“Ketamine”[MeSH Terms] OR “ketamine”[Title/Abstract]) AND (“Pediatric Dentistry”[MeSH Terms] OR “pediatric dental”[Title/Abstract] OR “children”[Title/Abstract] OR “pediatric”[Title/Abstract] OR “child”[MeSH Terms]) AND (“Conscious Sedation”[MeSH Terms] OR “sedation”[Title/Abstract] OR “procedural sedation”[Title/Abstract] OR “premedication”[Title/Abstract]) AND (“Randomized Controlled Trial”[Publication Type] OR “randomized”[Title/Abstract] OR “RCT”[Title/Abstract])). Equivalent strategies adapted for Scopus, Web of Science, and ScienceDirect are provided in Supplementary Table S1.

### 2.4. Screening and Study Selection

Two reviewers (AbdulRahman Alasmri, Ali Alhijab) independently screened titles and abstracts against the eligibility criteria. Full texts of potentially eligible articles were then independently assessed by both reviewers. Disagreements at both stages were resolved through discussion with a third reviewer (HJS).

Studies included in this review were selected following the PICO elements:

○ Participants

Children aged 2–14 years undergoing dental procedures including both procedural sedation and premedication prior to general anesthesia for dental treatment who received dexmedetomidine or ketamine.

○ Intervention

Administration of dexmedetomidine (any route) for sedation or premedication in pediatric dental procedures.

○ Comparisons

Administration of ketamine (any route) for sedation or premedication in pediatric dental procedures.

○ Outcome measures
○ Primary Outcome: Surrogate indicators of sedation quality including analgesic efficacy (assessed by FLACC scores where available), hemodynamic parameters (heart rate, SBP, DBP), oxygen saturation (SpO_2_), and recovery time. Note: Direct sedation efficacy outcomes (e.g., sedation scale scores, success rates) were assessed narratively where reported but could not be pooled quantitatively due to heterogeneity in measurement instruments across studies.
○ Secondary Outcome: Evaluation of the safety profiles of both drugs, including incidence of adverse effects, sedation onset time, duration of action, and recovery profiles.

### 2.5. Data Extraction

Data were extracted independently by two reviewers (AbdulRahman Alasmri, Ali Alhijab) using a pre-piloted standardized extraction form. Extracted variables included: study identifiers (first author, year, country), study design, sample size per group, patient demographics (age, weight), intervention details (drug, dose, route of administration, timing), outcome measures and their definitions, and numerical results (means, standard deviations, *p*-values). Disagreements were resolved by discussion with a third reviewer (HJS).

### 2.6. Inclusion Criteria

The inclusion criteria included RCT studies that compared the sedation of dexmedetomidine and ketamine for pediatric dental procedures. Studies with non-RCT designs were excluded. Studies evaluating dexmedetomidine without a ketamine comparator arm were also excluded. Other studies, such as editorials, letters to the editor, pilot studies, historical and literature reviews, in vitro studies, and descriptive studies (including case reports and case series), were also excluded.

### 2.7. Quality Assessment and the Risk of Bias

The risk of bias was assessed by two reviewers (AbdulRahman Alasmri, Ali Alhijab) independently using the Cochrane Risk of Bias tool (RoB 2.0) [11]. Five domains were evaluated for each included study: randomization process, deviations from intended interventions, missing outcome data, measurement of the outcome, and selection of the reported results. Each domain was judged as ‘Low Risk,’ ‘Some Concerns,’ or ‘High Risk.’ Two reviewers independently assessed all studies, and discrepancies were resolved by consensus.

### 2.8. Statistical Analysis

Mean differences (MDs) were calculated along with 95% confidence intervals (CIs) for various continuous outcomes, which include intraoperative analgesia, postoperative analgesia, heart rate, systolic blood pressure (SBP), diastolic blood pressure (DBP), oxygen saturation (SpO_2_), and recovery duration. A random-effects model using the restricted maximum-likelihood (REML) estimator with the Hartung–Knapp–Sidik–Jonkman (HKSJ) adjustment for confidence intervals was selected a priori to account for anticipated clinical and methodological heterogeneity across included studies, which differed in route of administration, drug dosage, and clinical setting (premedication vs. procedural sedation). The REML estimator provides less biased estimates of between-study variance than the method-of-moments approach, and the HKSJ adjustment produces confidence intervals based on a t-distribution, better reflecting uncertainty when the number of studies is small. This approach accounts for both within-study sampling error and between-study variance. Heterogeneity was quantified using the I^2^ statistic. Values of 0–40% may not represent important heterogeneity; 30–60% may represent moderate heterogeneity; 50–90% may represent substantial heterogeneity; and 75–100% may represent considerable heterogeneity, as recommended by the Cochrane Handbook [12]. In instances where significant heterogeneity is detected, we investigated potential contributors to this variability. A leave-one-out sensitivity analysis was performed for each pooled outcome, sequentially excluding each study to assess its influence on the summary estimate and to evaluate the robustness of pooled results. Since there are fewer than 10 studies, an analysis of publication bias was not conducted. Statistical significance was set at *p* < 0.05. All analyses were performed using Review Manager (RevMan Web), online version, The Cochrane Collaboration.

## 3. Results

A total of 1368 records were retrieved through systematic searches of electronic databases. After removing 1089 duplicate entries, 279 records remained for initial screening based on titles and abstracts. Of these, 268 records were excluded because they did not meet the inclusion criteria: 94 did not mention both interventions of interest, 80 were review or systematic review articles, 40 were case reports or case series, 32 were retrospective studies, 21 were animal studies, and 1 was a retracted publication.

**Figure 1:**
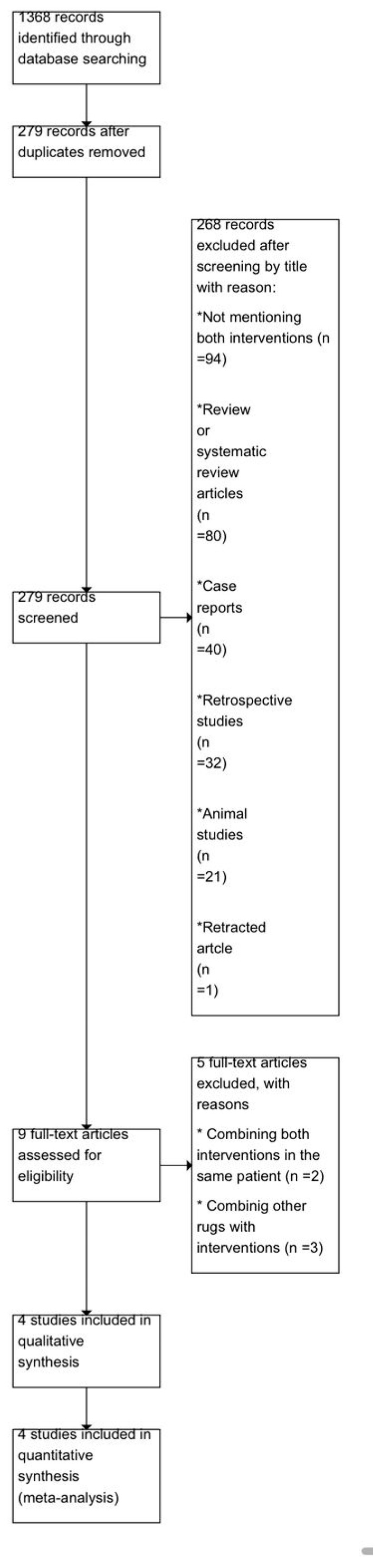
PRISMA 2020 flow chart.

The full texts of the remaining 9 articles were assessed for eligibility. Five studies were excluded for the following reasons: two studies used a design in which both interventions were combined in the same patient, precluding independent group comparisons, and three studies combined other pharmacologic agents with the target interventions, preventing isolated comparison of dexmedetomidine and ketamine. Four studies fulfilled all inclusion criteria and were included in the final qualitative and quantitative synthesis (Figure 1).

### 3.1. Study Characteristics

This review analyzes four randomized controlled trials comparing dexmedetomidine and ketamine for pediatric sedation or anesthesia. The trials involved 178 children (mean ages 6.5–9.1 years) from India, Egypt, and Syria (Table 1).

Surendar et al. [16] conducted a study using intranasal administration of dexmedetomidine (1 µg/kg) and ketamine (5 mg/kg) in children undergoing general anesthesia. A total of 42 participants were included (21 per group). The measured outcomes included onset and recovery times, intra- and postoperative analgesic use, sedation level, behavioral responses, hemodynamic parameters (RR, DBP, SBP), and success rates.

Zanaty et al. [14] conducted a study in Egypt involving 40 pediatric patients undergoing general anesthesia. Participants received nebulized dexmedetomidine (2 µg/kg) or nebulized ketamine (2 mg/kg) via intranasal nebulizer mask. The study assessed various factors, including ease of separation from parents, ease of venipuncture, acceptance of the face mask, sedation levels, recovery time, postoperative analgesia, respiratory rate (RR), and oxygen saturation (SpO_2_). These medications were administered 10–15 min before the induction of anesthesia. Hammadyeh et al. [13] (Syria, 2017–2019) studied the intravenous administration of dexmedetomidine (1 µg/kg) compared to ketamine (2 mg/kg) for procedural sedation in 40 children. They evaluated outcomes such as behavioral response, recovery time, and side effects. The groups’ ages and weights were similar. Singh et al. [12] (India) conducted a study involving 56 pediatric participants, with 28 individuals in each group receiving oral sedation via dexmedetomidine (5 µg/kg) or ketamine (8 mg/kg). The primary outcomes measured were the level of sedation and vital signs, including oxygen saturation (SpO_2_), pulse rate (PR), systolic and diastolic blood pressure (SBP, DBP), and respiratory rate (RR). Studies examined the use of intranasal, intravenous, and oral ketamine versus dexmedetomidine in pediatric patients (Table 2).

### 3.2. Risk of Bias

The risk-of-bias results are summarized in Table 3. Two of the included studies, Hammadyeh et al. [13] and Zanaty & El Metainy [14], were judged to have an overall low risk of bias. Both studies provided clear descriptions of randomization, ensured consistent administration of interventions, blinded the outcome assessments, and reported all pre-specified outcomes. Overall, they appeared methodologically sound and transparent.

The other two studies, Singh et al. [15] and Natarajan Surendar et al. [16], were rated as having some concerns. Although both claimed triple blinding, they did not demonstrate whether blinding was effectively maintained in practice. In addition, neither study registered a protocol or specified its outcomes prospectively, which raises the possibility of selective reporting. In Surendar et al., there were also uncertainties about whether the outcome assessors were adequately blinded, which added to the concerns in that domain.

**Figure 2:**
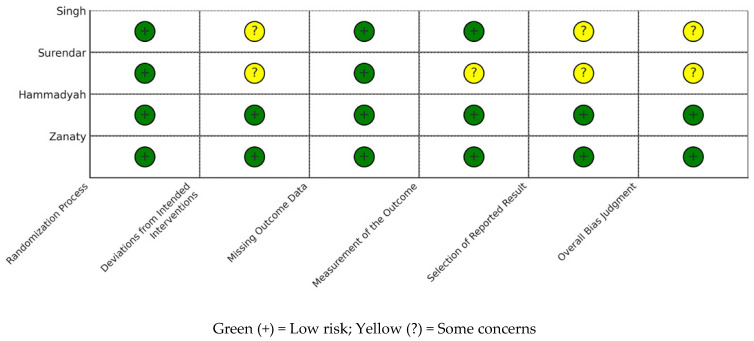
Risk-of-bias summary for included studies using the Cochrane RoB 2.0 tool.

Importantly, no studies were rated as having a high risk of bias, and all included studies reported their outcomes altogether, with no issues related to missing data (Figure 2; Table 3).

Given that two of four included studies had methodological concerns and the total evidence base comprised only 178 participants, these risk-of-bias findings contribute to the overall low certainty of evidence for all outcomes, as reflected in the GRADE assessment (Table 4).

### 3.3. Analgesia Outcomes

Intraoperative and postoperative analgesia are key indicators for effective sedation, ensuring adequate pain control, patient comfort, and optimal recovery.

Intraoperative Analgesia

**Figure 3:**
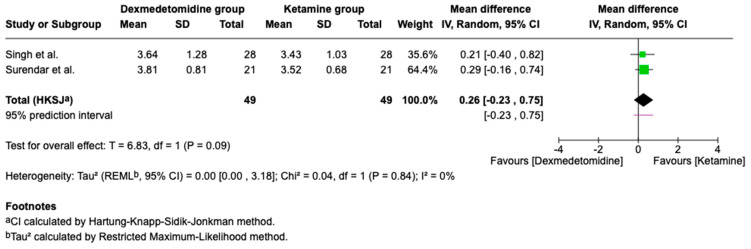
Forest plot comparing intraoperative analgesia of Dexmedetomidine vs. Ketamine (FLACC score) (higher scores indicate greater pain and thus poorer analgesia) [12,16].

Intraoperative FLACC scores (a behavioral pain scale for nonverbal patients assessing face, legs, activity, cry, and consolability) from two trials indicated no significant difference between ketamine and dexmedetomidine (Figure 3; Table 2).

### 3.4. Postoperative Analgesia

**Figure 4:**
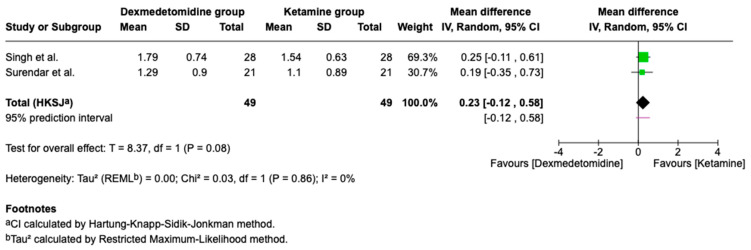
Forest plot comparing postoperative analgesia: Dexmedetomidine vs. Ketamine (FLACC score) (higher scores indicate greater pain and thus poorer analgesia) [12,16].

Combined postoperative FLACC scores from two studies demonstrated similar analgesic efficacy for both agents, with no statistically significant differences between the groups (Figure 4; Table 2).

### 3.5. Hemodynamic Outcomes

**Figure 5:**
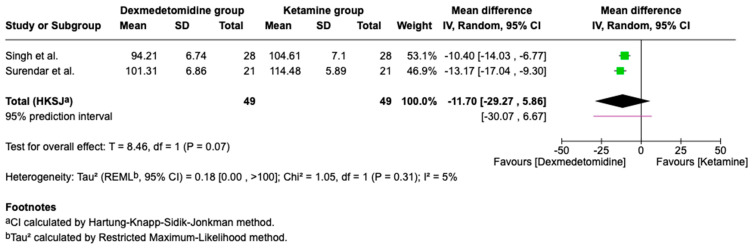
Forest plot comparing heart rate: Dexmedetomidine vs. Ketamine (beats/min) [12,16].

**Figure 6:**
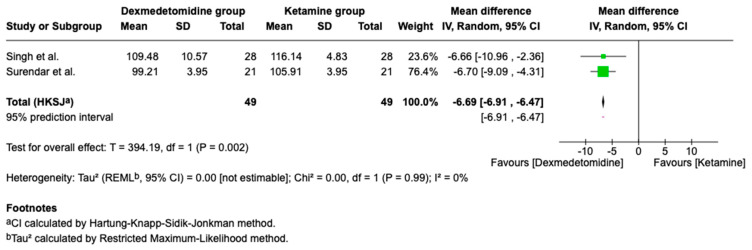
Forest plot for the meta-analysis comparing the effects of Dexmedetomidine and Ketamine on systolic blood pressure (mmHg) [12,16].

**Figure 7:**
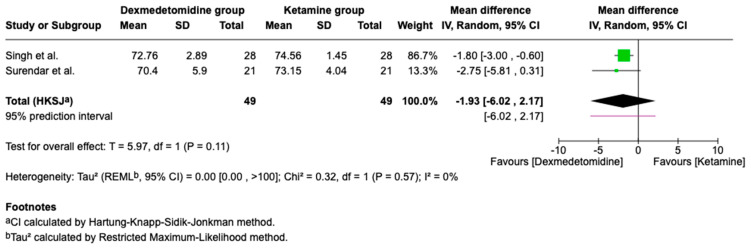
Forest plot comparing DBP of Dexmedetomidine and Ketamine (mmHg) [12,16].

Hemodynamic outcomes are key to assessing sedation safety, as they indicate how well a patient tolerates changes in heart rate and blood pressure during and after sedation. Pooled analyses of hemodynamic outcomes from two studies are presented below. While point estimates favored lower blood pressure and heart rate values with dexmedetomidine, the clinical significance and consistency of these findings should be interpreted in the context of the small number of contributing studies (Figure 5, Figure 6 and Figure 7).

### 3.6. Respiratory Parameters

**Figure 8:**
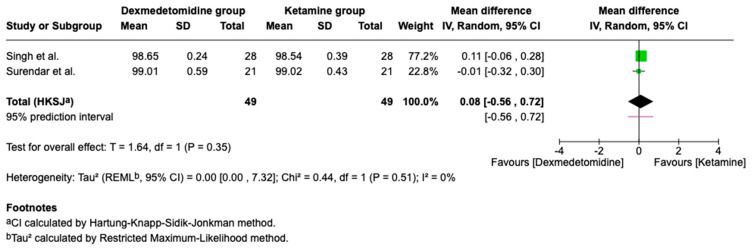
Meta-analysis forest plot of SpO_2_: Dexmedetomidine vs. Ketamine (%) [12,16].

Respiratory parameters help evaluate sedation safety by monitoring breathing and oxygenation, ensuring the patient remains stable throughout the procedure. Both groups in the two trials maintained SpO_2_ ≥ 98% without significant differences (Figure 8). Respiratory rates were also similar.

### 3.7. Recovery Profiles

**Figure 9:**
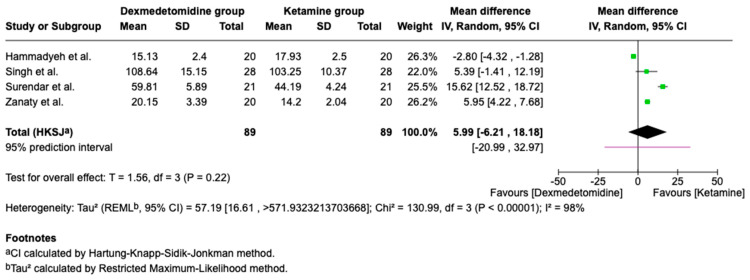
Meta-analysis of recovery time (min) in pediatric patients treated with dexmedetomidine compared with ketamine [12,13,14,16].

Recovery time is a clinically relevant outcome. Recovery time showed extreme heterogeneity across the four included studies (I^2^ = 98%), rendering the pooled estimate unreliable for clinical interpretation. This heterogeneity likely reflects differences in drug dosage (1–5 µg/kg for dexmedetomidine; 2–8 mg/kg for ketamine), route of administration (Intranasal (nebulized), intranasal (drops), intravenous, oral), and clinical context (premedication vs. procedural sedation). Therefore, this result should be interpreted descriptively rather than as evidence of a definitive difference between agents (Figure 9). All patients across studies resumed baseline functional status within clinically acceptable timeframes. Leave-one-out sensitivity analysis did not materially alter pooled estimates for any outcome, confirming the robustness of the findings.

### 3.8. Adverse Events

Adverse event reporting varied across the included studies. Hammadyeh et al. (2019) [13] was the only study that systematically monitored and reported adverse events as a predefined outcome, including intraoperative monitoring and 24 h postoperative telephone follow-up; no episodes of oxygen desaturation, hypotension, airway obstruction, bradycardia, or late complications were observed in either group. The remaining three studies (Surendar et al. 2014 [16]; Zanaty et al. 2015 [14]; Singh et al. 2014 [15]) reported no serious adverse events but did not describe structured adverse event monitoring protocols. Due to the absence of standardized adverse event reporting frameworks across studies, a formal quantitative synthesis of safety outcomes beyond hemodynamic and respiratory parameters was not feasible.

### 3.9. Narrative Synthesis of Sedation-Related Outcomes

The included studies reported several sedation-specific outcomes that could not be quantitatively pooled due to the use of different measurement instruments. Surendar et al. (2014) [16] assessed sedation level and success rate; Zanaty et al. (2015) [14] evaluated ease of separation from parents, ease of venipuncture, and face-mask acceptance; Hammadyeh et al. (2019) [13] reported behavioral response during the procedure; and Singh et al. (2014) [15] measured sedation level using a categorical scale. While individual study results suggested acceptable sedation with both agents, the lack of a common validated sedation instrument precludes quantitative synthesis of sedation efficacy as a primary endpoint.

The certainty of evidence for each outcome was assessed using the GRADE approach and summarized in a Summary of Findings table (Table 4).

## 4. Discussion

This systematic review and meta-analysis evaluated four randomized controlled trials comparing dexmedetomidine and ketamine for pediatric dental sedation. The pooled results did not demonstrate statistically significant differences between the two agents for most analyzed outcomes. Based on the limited evidence available, both agents appeared to provide adequate sedation-related outcomes, though the certainty of evidence was low across all comparisons.

The following pharmacological discussion provides mechanistic context for the observed findings but should not be interpreted as evidence derived from this meta-analysis. Dexmedetomidine is a selective α2-adrenoreceptor agonist that produces sedation resembling physiological sleep while allowing rapid arousal with stimulation. This property contributes to its favorable respiratory profile and minimal respiratory depression during procedural sedation [17,18]. These characteristics are particularly relevant in pediatric dental procedures where airway access may be partially limited.

Ketamine, in contrast, acts primarily as an N-methyl-D-aspartate (NMDA) receptor antagonist and produces dissociative sedation characterized by analgesia, amnesia, and preservation of airway reflexes [19]. In addition, ketamine stimulates the sympathetic nervous system, which can increase heart rate and blood pressure. These pharmacological properties explain the strong analgesic effect of ketamine as well as its cardiovascular stimulation.

The findings appear broadly aligned with existing literature; however, the limited number of included trials precludes definitive comparison. Dexmedetomidine has been reported to provide effective sedation with stable respiratory parameters and minimal respiratory depression [17,20]. Because of these characteristics, it has been increasingly used in pediatric medical and dental procedures.

Ketamine has also been widely used for pediatric sedation due to its strong analgesic properties and preservation of airway reflexes [19]. However, some studies have reported adverse effects such as increased salivation and cardiovascular stimulation [21,22]. Comparative studies suggest that dexmedetomidine may provide more stable sedation, whereas ketamine may offer stronger analgesic effects. The findings of the present meta-analysis are generally consistent with these observations, although direct comparisons remain limited due to differences in sedation protocols across studies.

Hemodynamic stability is an important factor when selecting sedative agents for pediatric dental procedures. Dexmedetomidine may decrease heart rate and blood pressure due to its central sympatholytic effects [23]. These changes are usually mild but may occasionally lead to bradycardia or hypotension in some patients. The cardiovascular response to dexmedetomidine has been described as biphasic, with an initial increase in blood pressure followed by a reduction in sympathetic activity and a decrease in arterial pressure [24].

Ketamine, on the other hand, produces cardiovascular stimulation through activation of the sympathetic nervous system, which typically increases heart rate and blood pressure [25]. In the studies included in this review, both drugs maintained stable oxygen saturation levels. Dexmedetomidine has been associated with minimal respiratory depression, which is an important advantage during pediatric sedation [17].

Recovery time is an important practical outcome in pediatric dental sedation because shorter recovery periods may improve treatment efficiency in clinical practice. In the present analysis, recovery time varied between studies comparing dexmedetomidine and ketamine. This variability may be related to differences in drug dosage, route of administration, and the use of additional sedative medications. Previous studies have also reported differences in recovery time associated with various sedation protocols [25].

Regarding postoperative pain, no statistically significant difference was observed between dexmedetomidine and ketamine in the included studies. Postoperative pain following extensive dental treatment has been reported to peak within the first 12 h after the procedure [25], and its intensity may increase with the extent of surgical intervention [26]. Some studies have also reported reduced early postoperative discomfort in patients receiving dexmedetomidine [27]. Overall, both agents appear to provide adequate postoperative pain control in pediatric dental procedures.

From a clinical perspective, both dexmedetomidine and ketamine appear to be suitable sedative agents for pediatric dental procedures. Dexmedetomidine may be advantageous when respiratory stability and calm sedation are priorities. Its minimal effect on respiration and its ability to reduce salivary secretions may be beneficial during dental procedures where airway control is important. Ketamine may be preferable when stronger analgesia is required or when cardiovascular stimulation is clinically advantageous. Therefore, the choice of sedative agent should be based on patient characteristics, procedure type, and clinician experience.

Several limitations should be considered when interpreting the findings of this meta-analysis. First, only four randomized controlled trials met the inclusion criteria, which limits the statistical power and generalizability of the results. Second, variations in sedation protocols, drug dosages, and routes of administration across studies may have introduced clinical heterogeneity. Third, the included trials had relatively small sample sizes, which may affect the precision of pooled estimates. The HKSJ adjustment for random-effects confidence intervals, when applied to meta-analyses with only two studies and near-zero heterogeneity, can produce narrow confidence intervals, as observed for systolic blood pressure (I^2^ = 0%). This is a recognized statistical property of the method and should be considered when interpreting the precision of pooled estimates for outcomes based on two studies. Some studies also raised methodological concerns regarding blinding and outcome reporting, which may introduce bias. The restricted geographical distribution of included trials (India, Egypt, Syria) may limit generalizability to other populations and healthcare systems. Also, the inclusion of studies with different sedation contexts (premedication before GA vs. chairside procedural sedation) introduces clinical indirectness. No included study reported long-term follow-up outcomes. The search was limited to four databases and English-language publications, which may not have captured all relevant unpublished or non-English-language trials.

Additionally, publication bias was not formally assessed because statistical tests for publication bias are considered unreliable when fewer than ten studies are included.

Furthermore, the comparative safety assessment in this review was limited to hemodynamic and respiratory parameters. Clinically relevant adverse events such as nausea, vomiting, excessive salivation, emergence agitation, and bradycardia were not consistently reported across the included studies. This inconsistency precludes definitive conclusions regarding the comparative safety profiles of the two agents and should be addressed in future trials through standardized adverse event monitoring and reporting.

The GRADE assessment rated the certainty of evidence as low for all outcomes. This rating reflects the combined impact of risk-of-bias concerns in two studies, imprecision due to small sample sizes, inconsistency (particularly for recovery time), and indirectness arising from heterogeneous sedation contexts and routes. Consequently, the findings of this review should be considered hypothesis-generating rather than definitive.

Further randomized controlled trials with larger sample sizes and standardized sedation protocols are needed to provide stronger evidence regarding the comparative effectiveness and safety of dexmedetomidine and ketamine in pediatric dental procedures. Future studies should also evaluate long-term outcomes, including recovery characteristics and patient-centered outcomes.

## 5. Conclusions

Current evidence from four small, clinically heterogeneous randomized controlled trials suggests that both dexmedetomidine and ketamine may be viable options for sedation or premedication in selected pediatric dental settings. The certainty of evidence is low across all outcomes, and no firm conclusion of superiority can be drawn for either agent. Both drugs demonstrated comparable profiles for analgesic outcomes, respiratory parameters, and hemodynamic variables within the limitations of the available data. The extreme heterogeneity observed for recovery time precludes reliable pooled conclusions for this outcome. Further multicenter randomized controlled trials with standardized sedation protocols, larger sample sizes, consistent outcome measures, and inclusion of geographically diverse populations are needed before definitive clinical recommendations can be made.

## Figures and Tables

**Table 1:** Characteristics of included studies.

Study	Country, Date	Treatment Modality	Route	No. of Participant	Dex.	Ket.	Timing	Measurements
					Dose, Mean Age Y (SD), Mean Weight kg (SD)	Dose, Mean Age Y (SD), Mean Weight kg (SD)		
Hammadyeh et al. [13]	Syria, 2017– 2019	Sedation	IV	Total = 40 G1 = 20 G2 = 20	1 μg∙kg^−1^, 9.1 (0.9), 14.9 (9.1)	2 mg∙kg^−1^, 8.9 (0.7), 15.2 (8.2)	NR	Behavior, recovery time, side effect
*Zanaty et al.* [14]	Egypt, NR	GA	*IN (Nebulized)*	Total = 40 G1 = 20 G2 = 20	2 μg∙kg^−1^, 3.55 (0.97), 17.38 (1.95)	2 mg∙kg^−1^, 3.37 (0.72), 16.88 (1.49)	10–15 min before GA	Ease of separation, Ease of venipuncture, face mask acceptance, level of sedation, recovery time, post op. analgesia, SpO_2_
Singh et al. [15]	India, NR	Sedation	Oral	Total = 56 G1 = 28 G2 = 28	5 μg∙kg^−1^, 6.82 (2.22), 16.61 (4.92)	8 mg∙kg^−1^, 6.54 (1.79), 16.89 (4.33)	NR	SpO_2_, PR, SBP, DBP, RR
Surendar et al. [16]	India, NR	GA	IN	Total = 42 G1 = 21 G2 = 21	1 μg∙kg^−1^, 7.76 (2.26), 18.75 (4.17)	5 mg∙kg^−1^, 6.71 (2.3), 16.52 (3.87)	NR	RR, DBP, SBP, Sedation level, Behavior, Onset time, Recovery time, Success rate, Intra-post op. Analgesic

Dex. = Dexmedetomidine; (SD) = Standard deviation; Ket. = Ketamine; GA = General anesthesia; IV = Intravenous; IN = Intranasal; Neb = Nebulized; G1 = Group 1; G2 = Group 2; NR = Not reported; Y = Year; kg = kilogram; SBP = Systolic blood pressure; DBP = Diastolic blood pressure; PR = Pulse Rate; RR = Respiratory rate; Intra-op. = Intraoperative analgesic; Post op. analgesic = Postoperative analgesic; SpO_2_ = O_2_ saturation.

**Table 2:** Studies included in the meta-analysis and their measurements.

Hammadyeh et al. [13]			Zanaty et al. [14]			Surendar et al. [16]			Singh et al. [12]				
*p* Value	Ket. Mean (SD)	Dex. Mean (SD)	*p* Value	Ket. Mean (SD)	Dex. Mean (SD)	*p* Value	Ket. Mean (SD)	Dex. Mean (SD)	*p* Value	Ket. Mean (SD)	Dex. Mean (SD)	Unit	Tools
NR	NR	NR	NR	NR	NR	0.475	99.02 (0.43)	99.01 (0.59)	0.2	98.54 (0.39)	98.65 (0.24)	%	SpO_2_
NR	NR	NR	NR	NR	NR	<0.001	114.48 (5.89)	101.31 (6.86)	<0.00	104.61 (7.10)	94.21 (6.74)	beats/min	PR
NR	NR	NR	NR	NR	NR	<0.001	105.91 (3.95)	99.21 (8.55)	<0.001	116.14 (4.83)	109.48 (10.57)	mmHg	SBP
NR	NR	NR	NR	NR	NR	0.04	73.15 (4.04)	70.40 (5.90)	<0.001	74.56 (1.45)	72.76 (2.89)	mmHg	DBP
<0.001	17.93 (2.5)	15.13 (2.4)	<0.001	14.20 (2.04)	20.15 (3.39)	<0.001	44.19 (4.24)	59.81 (5.89)	0.12	103.25 (10.37)	108.64 (15.15)	min	Recovery time
NR	NR	NR	NR	NR	NR	0.1	3.52 (0.68)	3.81 (0.81)	0.5	3.43 (1.03)	3.64 (1.28)	FLACC	Intra-op. analgesic
												Score	
NR	NR	NR	NR	NR	NR	0.24	1.10 (0.89)	1.29 (0.90)	0.17	1.54 (0.63)	1.79 (0.74)	FLACC	Post op. analgesic
												Score	

Dex. = Dexmedetomidine; (SD) = Standard deviation; Ket. = Ketamine; SpO_2_ = O_2_ Saturation; PR = Pulse Rate per minute; SBP = Systolic blood pressure; DBP = Diastolic blood pressure; Intra-op. = Intraoperative analgesic; Post op. analgesic = Postoperative analgesic; NR = Not reported; FLACC = Face, Legs, Activity, Cry, Consolability scale.

**Table 3:** Risk-of-Bias Assessment Using the Cochrane RoB 2.0 Tool for Included Randomized Controlled Trials.

Study	Randomization Process	Deviations from Intended Interventions	Missing Outcome Data	Measurement of the Outcome	Selection of the Reported Result	Overall Risk of Bias
Hammadyeh et al., 2019 [13]	Low Risk	Low Risk	Low Risk	Low Risk	Low Risk	Low Risk
Zanaty et al., 2015 [14]	Low Risk	Low Risk	Low Risk	Low Risk	Low Risk	Low Risk
Singh et al., 2014 [15]	Low Risk	Some Concerns	Low Risk	Low Risk	Some Concerns	Some Concerns
Surendar et al., 2014 [16]	Low Risk	Some Concerns	Low Risk	Some Concerns	Some Concerns	Some Concerns

**Table 4:** Summary of Findings and Certainty of Evidence (GRADE).

Outcome	№. Participants (Studies)	Certainty (GRADE)	Mean Difference [95% CI]	*p* Value	Reasons for Downgrading
Intraoperative analgesia (FLACC score)	98 (2 RCTs)	⊕⊕⊖⊖ Low	MD 0.26 higher (−0.23 to 0.75 higher)	0.09	a, b, d
Postoperative analgesia (FLACC score)	98 (2 RCTs)	⊕⊕⊖⊖ Low	MD 0.23 higher (−0.12 to 0.58 higher)	0.08	a, b, d
Recovery time (min)	178 (4 RCTs)	⊕⊕⊖⊖ Low	MD 5.99 higher (−6.21 to 18.18 higher)	0.22	a, b, c, d
Heart rate (beats/min)	98 (2 RCTs)	⊕⊕⊖⊖ Low	MD −11.70 (−29.27 to 5.86)	0.07	a, b, d
Systolic blood pressure (mmHg)	98 (2 RCTs)	⊕⊕⊖⊖ Low	MD −6.69 (−6.91 to −6.47)	0.002	a, b, d
Diastolic blood pressure (mmHg)	98 (2 RCTs)	⊕⊕⊖⊖ Low	MD −1.93 (−6.02 to 2.17)	0.11	a, b, d
Oxygen saturation (SpO_2_, %)	98 (2 RCTs)	⊕⊕⊖⊖ Low	MD 0.08 (−0.56 to 0.72)	0.35	a, b, d

Reasons for downgrading: a. Risk of bias: Two of four studies had some concerns regarding blinding maintenance and absence of prospective protocol registration. b. Imprecision: Small total sample sizes (n = 98 for two-study outcomes; n = 178 for recovery time), with wide confidence intervals crossing the null for most outcomes. c. Inconsistency: Applicable to recovery time only (I^2^ = 98%), indicating extreme between-study variability. d. Indirectness: Mixed sedation contexts (intranasal nebulized premedication before GA, intravenous procedural sedation, oral sedation) and heterogeneous dosing protocols, ⊕⊕⊖⊖ = Low certainty of evidence (GRADE).

## Data Availability

The dataset is available on request from the authors. The data are not publicly available due to confidentiality considerations.

## Supplementary Materials

The following supporting information can be downloaded at: https://www.mdpi.com/article/10.3390/children13040558/s1, Supplementary Table S1: Database-Specific Search Strategies; Supplementary Table S2: Risk of Bias Assessment (RoB 2.0) Domain-Level Justification for Included Studies. Supplementary Table S3. Full-Text Articles Excluded with Reasons for Exclusion [7,28,29,30,31].

## Abbreviations

The following abbreviations are used in this manuscript:

Dex	Dexmedetomidine
SD	Standard deviation
Ket.	Ketamine
GA	General anesthesia
IV	Intravenous
IN	Intranasal
Neb	Nebulized
NR	Not Reported
Y	Year
kg	Kilogram
SBP	Systolic blood pressure
DBP	Diastolic blood pressure
RR	Respiratory rate
PR	Pulse Rate per minute
Intra-op. analgesic	Intraoperative analgesic
Post op. analgesic	Postoperative analgesic
SpO_2_	O_2_ saturation
FLACC	Face, Legs, Activity, Cry, Consolability scale.
