# Supplementary material for: The Efficacy of Dexmedetomidine Versus Ketamine for Sedation in Pediatric Dental Procedures: A Systematic Review and Meta-Analysis
Source: Children (Basel). 2026 Apr 17;13(4):558. doi: 10.3390/children13040558 (PMC13114709; doi:10.3390/children13040558)
Supplement: Supplementary file 1 [file children-13-00558-s001.zip › 2026.03.26_Supplementary Table S1.pdf]

Supplementary Table S1. Complete Database-Specific Search Strategies

Database	Full Search String	Filters / Limits Applied	Date Executed
**PubMed / MEDLINE**	(("Dexmedetomidine"[MeSH Terms] OR "dexmedetomidine"[Title/Abstract] OR "precedex"[Title/Abstract]) AND ("Ketamine"[MeSH Terms] OR "ketamine"[Title/Abstract]) AND ("Pediatric Dentistry"[MeSH Terms] OR "pediatric dental"[Title/Abstract] OR "Child"[MeSH Terms] OR "children"[Title/Abstract] OR "paediatric"[Title/Abstract] OR "infant"[Title/Abstract]) AND ("Conscious Sedation"[MeSH Terms] OR "sedation"[Title/Abstract] OR "procedural sedation"[Title/Abstract] OR "premedication"[Title/Abstract] OR "Deep Sedation"[MeSH Terms]) AND ("Randomized Controlled Trial"[Publication Type] OR "randomized"[Title/Abstract] OR "randomised"[Title/Abstract] OR "RCT"[Title/Abstract] OR "controlled trial"[Title/Abstract]))	Publication date: 1990/01/01 to 2026/01/15 Species: Humans Language: English	January 15, 2026
**Scopus**	TITLE-ABS-KEY ((dexmedetomidine OR precedex) AND (ketamine) AND ("pediatric dentistry" OR "pediatric dental" OR child OR children OR paediatric OR infant) AND (sedation OR "procedural sedation" OR "conscious sedation" OR premedication OR "deep sedation") AND (randomized OR randomised OR "controlled trial" OR RCT))	Date range: 1990–2026 Document type: Article Language: English Subject area: Medicine, Dentistry	January 15, 2026
**Web of Science (Core Collection)**	TS = ((dexmedetomidine OR precedex) AND (ketamine) AND ("pediatric dentistry" OR "pediatric dental" OR child* OR paediatric OR infant) AND (sedation OR "procedural sedation" OR "conscious sedation" OR premedication OR "deep sedation") AND (randomized OR randomised OR "controlled trial" OR RCT))	Timespan: 1990–2026 Document type: Article Language: English Indexes: SCI-EXPANDED, SSCI	January 15, 2026
**ScienceDirect**	Title, abstract, keywords: (dexmedetomidine OR precedex) AND (ketamine) AND ("pediatric dental" OR "pediatric dentistry" OR children OR child OR paediatric) AND (sedation OR "procedural sedation" OR premedication) AND (randomized OR randomised OR "controlled trial")	Year: 1990–2026 Article type: Research articles Subject: Dentistry, Medicine	January 15, 2026
**Google Scholar (supplementary hand search)**	dexmedetomidine ketamine "pediatric dental" sedation randomized	First 100 results screened Date range: 1990–2026	January 15, 2026

Notes:

1. MeSH = Medical Subject Headings (applicable to PubMed only). Scopus, Web of Science, and ScienceDirect use free-text equivalents.
2. Reference lists of all included studies and relevant review articles were manually screened for additional eligible studies.
3. Duplicate records were identified and removed using EndNote reference manager (version 9, Niles Software, USA).
4. The search date range (1990–2026) was selected because dexmedetomidine was first introduced into clinical practice in 1999; the broad starting date was intended to capture any early comparative investigations.
