# Supplementary material for: The Efficacy of Dexmedetomidine Versus Ketamine for Sedation in Pediatric Dental Procedures: A Systematic Review and Meta-Analysis
Source: Children (Basel). 2026 Apr 17;13(4):558. doi: 10.3390/children13040558 (PMC13114709; doi:10.3390/children13040558)
Supplement: Supplementary file 1 [file children-13-00558-s001.zip › Supplementary Table S2.pdf]

Supplementary Table S2. Risk of Bias Assessment (RoB 2.0): Domain-Level Judgments and Rationale for Included Studies

Study	D1: Randomization Process	D2: Deviations from Intended Interventions	D3: Missing Outcome Data	D4: Measurement of the Outcome	D5: Selection of the Reported Result	Overall
**Zanaty et al. (2015)**	**Low Risk**<br>Computer generated randomization with sealed opaque envelopes.<br><br>Adequate allocation concealment described.	**Low Risk**<br>Double blinded study. Intervention preparation and administration performed by personnel not involved in outcome assessment.<br>No evidence of unblinding.	**Low Risk**<br>All randomized participants completed the study.<br>No dropouts or missing data reported.	**Low Risk**<br>Outcome assessors were blinded to group allocation.<br>Validated and objective measurement tools used (sedation scales, SpO_2 monitors).	**Low Risk**<br>All outcomes listed in the methods section were reported in the results.<br>No evidence of selective reporting.	**Low Risk**
**Hammadyeh et al. (2019)**	**Low Risk**<br>Randomization performed using a computer generated sequence.<br><br>Allocation concealment maintained through numbered sealed envelopes.	**Low Risk**<br>Study described as double-blinded. Drugs prepared by an independent pharmacist in identical syringes.<br>No protocol deviations reported.	**Low Risk**<br>Complete follow-up achieved for all 40 participants.<br>No attrition or missing values.	**Low Risk**<br>Outcomes assessed by a blinded investigator using standardized instruments. Behavioral assessment and vital sign monitoring performed consistently across groups.	**Low Risk**<br>Study prospectively described all outcomes.<br>All listed endpoints were reported with complete data.	**Low Risk**
**Singh et al. (2014)**	**Low Risk**<br>Randomization described using a computer generated random number table.<br><br>Group allocation appeared adequate.	**Some Concerns**<br>Study claimed triple blinding but did not describe the specific method by which blinding was maintained after allocation.<br>Unable to confirm effectiveness of blinding.	**Low Risk**<br>All 56 randomized participants completed the study.<br>No missing outcome data.	**Low Risk**<br>Vital signs recorded using calibrated monitors.<br>Sedation assessed using a categorical scale by designated assessors.	**Some Concerns**<br>No prospective protocol registration identified.<br>Outcomes were not pre-specified in a publicly accessible registry, raising the possibility of selective outcome reporting.	**Some Concerns**
**Surendar et al. (2014)**	**Low Risk**<br>Randomization performed using a random number table.<br><br>Allocation to groups appeared adequate with no baseline imbalances.	**Some Concerns**<br>Study described triple blinding but did not provide sufficient detail on how blinding was operationalized for intranasal drug administration).<br>Effectiveness of blinding uncertain.	**Low Risk**<br>All 42 participants completed the trial.<br>Complete data reported for all outcomes.	**Some Concerns**<br>Uncertainty regarding whether outcome assessors were adequately blinded to group assignment. The study did not explicitly confirm that assessors of behavioral and sedation outcomes were unaware of the allocated intervention.	**Some Concerns**<br>No prospective protocol registration identified.<br>The study did not reference a pre-registered analysis plan, limiting the ability to assess whether all intended outcomes were reported.	**Some Concerns**

Abbreviations and Notes:

D1–D5 = Domains 1–5 of the Cochrane Risk of Bias 2.0 tool (Sterne et al., 2019)(1).

Low Risk = the study is judged to be at low risk of bias for this domain.

Some Concerns = there is some concern about the risk of bias for this domain, but insufficient evidence to judge as high risk.

Overall judgment follows the RoB 2.0 algorithm: if any domain is rated "Some Concerns," the overall judgment is "Some Concerns" unless multiple domains raise concerns, in which case "High Risk" may apply.

SpO_2 = peripheral oxygen saturation.

References:

1. Sterne JAC, Savović J, Page MJ, Elbers RG, Blencowe NS, Boutron I, et al. RoB 2: a revised tool for assessing risk of bias in randomised trials. Bmj. 2019.
