# Supplementary material for: The Efficacy of Dexmedetomidine Versus Ketamine for Sedation in Pediatric Dental Procedures: A Systematic Review and Meta-Analysis
Source: Children (Basel). 2026 Apr 17;13(4):558. doi: 10.3390/children13040558 (PMC13114709; doi:10.3390/children13040558)
Supplement: Supplementary file 1 [file children-13-00558-s001.zip › Supplementary Table S3.pdf]

Supplementary Table S3. Full-Text Articles Excluded with Reasons for Exclusion

Manuscript: The Efficacy of Dexmedetomidine versus Ketamine for Sedation in Pediatric Dental Procedures: A Systematic Review and Meta-Analysis

A total of 9 full-text articles were assessed for eligibility. Five were excluded for the reasons listed below. Four studies met all inclusion criteria and were included in the final synthesis.

#	Study (Author, Year)	Title	Reason for Exclusion
1	**Jaikaria, A.et al., 2018**(1)	A Comparison of Oral Midazolam-ketamine, Dexmedetomidine-fentanyl, and Dexmedetomidine-ketamine Combinations as Sedative Agents in Pediatric Dentistry: A Triple-Blinded Randomized Controlled Trial	Due to combining other drugs with interventions
2	**Malhotra, P. U.et al., 2016**(2)	Comparative evaluation of dexmedetomidine and midazolam-ketamine combination as sedative agents in pediatric dentistry: A double-blinded randomized controlled trial	Due to combining other drugs with interventions
3	**Joshi, A. B. et al., 2020(3)**	To Compare the Efficacy of Two Intravenous Combinations of Drugs Ketamine-Propofol vs Ketamine-Dexmedetomidine for Sedation in Children Undergoing Dental Treatment	Due to combining other drugs with interventions
4	**Baroni, DA et al., 2023(4)**	Nociception associated with pain/distress in young children sedated for dental treatment: a clinical study with objective and subjective measures	Due to both interventions on same patient
5	**Haider, K et al., 2022(5)**	A double-blind randomized controlled trial to compare the safety and efficacy of dexmedetomidine alone and in combination with ketamine in uncooperative and anxious paediatric dental patients requiring pulpectomy	Due to both interventions on same patient

Notes:

1. Exclusion categories: Three studies were excluded due to combining other drugs with interventions (#1–3); two studies were excluded due to both interventions on same patient (#4–5).
2. These numbers are consistent with the PRISMA flow diagram (Figure 1): 9 full-text articles assessed – 5 excluded = 4 included studies.

References:

1. Jaikaria A, Thakur S, Singhal P, Chauhan D, Jayam C, Syal K. A Comparison of Oral Midazolam-ketamine, Dexmedetomidine-fentanyl, and Dexmedetomidine-ketamine Combinations as Sedative Agents in Pediatric Dentistry: A Triple-Blinded Randomized Controlled Trial. Contemporary clinical dentistry. 2018;9(Suppl 2):S197-s203.
2. Malhotra PU, Thakur S, Singhal P, Chauhan D, Jayam C, Sood R, et al. Comparative evaluation of dexmedetomidine and midazolam-ketamine combination as sedative agents in pediatric dentistry: A double-blinded randomized controlled trial. Contemporary clinical dentistry. 2016;7(2):186-92.
3. Joshi AB, Shankaranarayan UR, Hegde A, Manju R. To Compare the Efficacy of Two Intravenous Combinations of Drugs Ketamine-Propofol vs Ketamine-Dexmedetomidine for Sedation in Children Undergoing Dental Treatment. International journal of clinical pediatric dentistry. 2020;13(5):529-35.
4. Baroni D, Corrêa-Faria P, Anabuki A, Abreu L, Costa LJEAoPD. Nociception associated with pain/distress in young children sedated for dental treatment: a clinical study with objective and subjective measures. 2023;24(2):255-62.
5. Haider K, Mittal N, Srivastava B, Gupta NJEAoPD. A double-blind randomized controlled trial to compare the safety and efficacy of dexmedetomidine alone and in combination with ketamine in uncooperative and anxious paediatric dental patients requiring pulpectomy. 2022;23(3):465-73.
